# Novel Approach to Skin Anti-Aging: Boosting Pharmacological Effects of Exogenous Nicotinamide Adenine Dinucleotide (NAD^+^) by Synergistic Inhibition of CD38 Expression

**DOI:** 10.3390/cells13211799

**Published:** 2024-10-30

**Authors:** Seongsu Kang, Jiwon Park, Zhihong Cheng, Sanghyun Ye, Seung-Hyun Jun, Nae-Gyu Kang

**Affiliations:** 1 LG Household and Health Care R&D Center, Seoul 07795, Republic of Korea; franck.kang@lghnh.com (S.K.); jwon1222@lghnh.com (J.P.); shye123@lghnh.com (S.Y.); 2 Department of Natural Medicine, School of Pharmacy, Fudan University, Shanghai 201203, China; chengzhh@fudan.edu.cn

**Keywords:** nicotinamide adenine dinucleotide (NAD^+^), quercetin, enoxolone, anti-aging, topical application, CD38

## Abstract

Nicotinamide adenine dinucleotide (NAD^+^) is indispensable for the regulation of biological metabolism. Previous studies have revealed its role in aging and degenerative diseases, while crucially showing that supplementation with NAD^+^ or its precursors could ameliorate or reverse the progression of aging. Despite extensive evidence for the role and action of NAD^+^ in aging, its pharmacological activity on the skin, or even its mechanism, has not been elucidated. In this study, we established a novel approach to effectively utilize NAD^+^ for skin anti-aging by enhancing the pharmacological efficacy of exogenous NAD^+^ using a phytochemical complex consisting of quercetin, and enoxolone through inhibition of CD38. Through the comprehensive in vitro experiments based on human fibroblasts, we observed that exogenous NAD^+^ could exert protective effects against both extrinsic aging induced by ultraviolet light exposure and intrinsic aging. Additionally, we found that its effects were significantly boosted by quercetin and enoxolone. In this in-depth study, we demonstrated that these beneficial effects are mediated by improved sirtuin activation, autophagy, and mitochondrial functionality. Our approach is expected to verify the applicability of the topical application of NAD^+^ and offer more effective solutions for the unmet needs of patients and consumers who demand more effective anti-aging effects.

## 1. Introduction

Nicotinamide adenine dinucleotide (NAD^+^), one of the most indispensable biological molecules, has been intensively studied in recent decades because of its importance in cellular metabolism. NAD^+^ participates in redox reactions by transferring electrons between NAD^+^ (the oxidized form of NAD) and NADH (the reduced form of NAD). It acts as a cofactor for enzyme reactions involving poly(ADP-ribose) polymerases (PARPs), sirtuins, and NAD glycohydrolases (CD38 and CD157) [1,2]. Due to its biological importance, NAD^+^ biosynthesis and its related metabolic pathways are highly conserved between yeast and vertebrates [3]. NAD^+^ biosynthesis is maintained through three pathways: de novo synthesis, which converts tryptophan into quinolinic acid (QA); NAD^+^, nicotinamide (NAM)/nicotinic acid (NA) salvage; and nicotinamide riboside (NR) salvage [4].

Despite its biological significance, the precise roles of NAD^+^ and its downstream targets remain unclear. Studies over the past two decades have highlighted its roles and actions in the aging process [5,6]. They have demonstrated an age-dependent decline in NAD^+^ levels and their association with the hallmarks of aging and age-related diseases. The decline in cellular NAD^+^ levels presumably results from various metabolic mechanisms: (1) Decreased NAD^+^ biosynthesis caused by decreased expression of nicotinamide phosphoribosyltransferase (NAMPT) [7], (2) increased NAD^+^ degradation or salvage mediated by the upregulation of CD38 [8], and (3) excessive consumption of NAD^+^ to counteract degenerative metabolic conditions and inflammations [9,10]. Although it is still debatable whether NAD^+^ decline is the cause or the result of aging, various forms of NAD^+^ supplementation have been demonstrated to effectively ameliorate, and even reverse, the hallmarks of aging. Taking it a step further, recent advances in understanding the relationship between NAD^+^ level and lifespan verified that boosting NAD^+^ levels can extend the lifespans of several organisms, including budding yeast [11], *Caenorhabditis elegans* [12,13], fruit flies (Drosophilidae) [14], and mice [15,16].

To date, the effects of NAD^+^ supplementation on humans have been marginally studied. Boosting systemic NAD^+^ by injection or oral administration of NAD^+^ precursors can delay and ameliorate the progress and symptoms in models of degenerative disease. For example, NAD^+^ restoration in humans improves neurodegenerative diseases [17,18], mitochondrial function [19], muscle function [20], and glucose metabolism, exerting effects such as enhanced insulin sensitivity [21] and body composition [22] under aging-related pathological conditions. However, the detailed mechanisms and actions remain unclear, and their ultimate effects on human lifespan are uncertain.

Similarly, the effects of NAD^+^ supplementation on human skin are not fully understood. A limited number of studies have been conducted to verify the in vitro efficacy of NAD^+^ precursors on skin cells, including NAM [23,24], NR (tested against primary fibroblasts from dyskeratosis congenita patients) [25], and nicotinamide mononucleotide (NMN, orally co-administered with *Lactobacillus* ferment to mice) [26]. However, their experimental observations were limited, and no study has investigated the effects of NAD^+^ applied to skin cells. Although NAD^+^ undoubtedly both serves as a key index of aging and ameliorates the hallmarks of aging, clearer evidence and investigations into the pharmacological efficacy of NAD^+^ on the skin are necessary for further industrial applications.

In this study, we not only demonstrated the pharmacological efficacy of NAD^+^ on human fibroblasts but also developed an approach to enhance this efficacy mediated by the phytochemical complex. Quercetin and enoxolone synergistically enhanced the bioavailability of exogenous NAD^+^ and consequently elevated cellular NAD^+^ levels by effectively inhibiting CD38 expression in fibroblasts.

We investigated the protective effects of NAD^+^ or NAD^+^ with a boosting complex (a complex of quercetin and enoxolone) on both photoaging and intrinsic aging and also underlying molecular actions and mechanisms.

Supplementing the skin with NAD^+^, also by topical application, is not widely used in industries at present. Our research, investigating the benefits of NAD^+^ on the skin and enhancing our understanding of its pharmacological effects, will provide new insights and clues for current research into NAD^+^, which remains a topic of vigorous discussion.

## 2. Materials and Methods

### 2.1. Cell Preparation

As described in the previous research, both intrinsic and extrinsic skin aging are highly associated with pathophysiologic changes in the dermis through enhanced release of the senescence-associated secretory phenotype (SASP), development of irreversible proliferation arrest, and degradation of extracellular matrix (ECM) by elevated proteolytic enzymes [27,28]. For this reason, the fibroblasts were targeted in this study.

Fibroblasts (HS68) were purchased from ATCC (American Type Culture Collection, Manassas, VA, USA) and cultured with DMEM (Gibco, Waltham, MA, USA) supplemented with 10% FBS (Gibco, Waltham, MA, USA) and penicillin–streptomycin (Gibco, Waltham, MA, USA) at 37 °C with 5% CO_2_. To exclude the effect of intrinsic aging for consistency of in vitro experiments, only fibroblasts with passages 7–10 were used. Before day one of the experiments, cells were detached from the cell culture flask using trypsin (Trypsin–EDTA (0.25%), Thermo Scientific, Waltham, MA, USA) and seeded in 12-well cell culture plates (SPL life science, Pocheon, Republic of Korea). When test materials were added to cells in the subsequent cell experiments, DMEM containing 1% FBS was used to exclude the serum-free effect of NAD^+^ synthesis and autophagy.

RAW264.7 mouse cells were obtained from Meilunbio^®^ (Dalian Meilun Biotechnology Co., Ltd., Dalian, China) and cultured in DMEM at 37 °C with 5% CO_2_.

Quercetin and enoxolone were purchased from Aladdin (Los Angeles, CA, USA). Quercetin is a plant flavonoid that is widely distributed in nature and found in many fruits and vegetables. Enoxolone, also called glycyrrhetinic acid, is a pentacyclic triterpenoid derivative obtained from the hydrolysis of glycyrrhizic acid from licorice. Both phytochemicals are used in cosmetics and dietary supplements and to treat specific pathological conditions owing to their anti-inflammatory or protective effects against oxidative stress [29,30].

### 2.2. UV-Induced Photoaging Model

On the day of the experiments, the cell culture media was aspirated and DPBS (Dulbecco’s phosphate buffered saline, Solbio, Seoul, Republic of Korea) was added to each well. Then, the cell culture was irradiated with ultraviolet light using an ultraviolet irradiator (Bio-sun, Vilber Lourmat, Collégien, France). The distance between the UV light source and the cell monolayer was 10 cm. The detailed irradiation conditions are 15 mJ/cm^2^ of 254 nm narrow-band UVA and 30 mJ/cm^2^ of 312 nm narrow-band UVB. The PBS was removed, and DMEM with test material was added to the wells. After 24 h, gene expression was analyzed. For protein expression analysis based on ELISA (Enzyme-linked Immunosorbent assay), supernatant media were collected after 48 h.

### 2.3. Analysis of Gene and Protein Expression

For analysis of gene expression, RT-qPCR (Real-time Polymerase Chain Reaction) was performed. RNA was extracted from fibroblasts using AccuPrep^®^ Universal RNA Extraction Kit (Bioneer, Daejeon, Republic of Korea). Total RNA (0.5 μg) was reverse-transcribed into cDNA using a cDNA synthesis kit (TOPscript™ cDNA Synthesis Kit (dN6 Mix), Enzynomics, Daejeon, Republic of Korea) following the manufacturer’s protocol in a Veriti 96-Well Thermal Cycler (Applied Biosystems, Waltham, MA, USA). Amplifications of cDNA and RT-qPCR were performed using the StepOnePlus™ RT-PCR system (Applied Biosystems, Waltham, MA, USA) and RT-qPCR kit (AccuPower^®^ GreenStar™ RT-qPCR PreMix, Bioneer, Republic of Korea) according to the protocol developed by the manufacturer. The following thermocycling conditions were used for qPCR: a total of 30 cycles at 95 °C for 45 s, 60 °C for 1 min, and 72 °C for 45 s. The primer sequences are presented in Supplementary Table S1.

For quantitative analysis of MMP-1 protein expression, ELISA was performed. The ELISA kit was purchased from R&D systems (Minneapolis, MN, USA).

### 2.4. Measurement of Cellular NAD^+^ and ATP

A commercially available assay kit (PicoSens™ NAD/NADH Assay Kit, Biomax, Guri-si, Republic of Korea) was used to quantify cellular NAD^+^ according to the manufacturer’s protocol. Briefly, test materials (NAD^+^, quercetin, enoxolone, and their combination) were applied to fibroblasts for 24 h, and the supernatant of the cell culture was removed. Cells were washed three times using PBS and lysed using the lysis buffer provided with the kit. Lysed cell samples were centrifuged and their supernatants were transferred into two microtubes, one for measuring total NAD (NAD + NADH) and another for measuring NADH. Microtubes for measuring NADH were heated for 30 min at 60 °C. According to the manufacturer’s guidelines, total NAD and NADH were quantified using enzymatic reactions. The amount of NAD^+^ was calculated by subtracting the NADH amount from the total NAD^+^.

Cellular ATP was measured by a commercially available assay kit (PicoSens™ ATP Assay Kit, Biomax, Republic of Korea). Similar to NAD^+^ measurement, cells were lysed and ATP was quantified using an enzymatic reaction.

### 2.5. Analysis of NAD^+^ Metabolites Using HPLC

NAD^+^ metabolites such as NAM, NR, NMN, NA (Nicotinic acid), and NaMN (Nicotinate mononucleotide) were analyzed using high-performance liquid chromatography (HPLC) based on a previous study [31]. Briefly, 0.05 M phosphate buffer (pH 7.0) reconstituted with potassium phosphate monobasic, potassium phosphate dibasic, and methanol were used as HPLC buffers. The HPLC was run at a flow rate of 1 mL/min with 100% buffer A (0.05 M Phosphate Buffer) from 0–5 min, a linear gradient to 95% buffer A/5% buffer B (100% methanol), a linear gradient to 85% buffer A/15% buffer B, a linear gradient to 85% buffer A/15% buffer B, a linear gradient to 100% buffer A, and 100% buffer A for 10 min. The HPLC-grade reference chemicals (0.1 ppm, 1 ppm, and 10 ppm) were individually analyzed as demonstrated in the previous paper [31]. Based on each reference spectrum, NAD^+^ metabolites in cell lysates were characterized. Cell lysates prepared by Igepal CA-630 (Sigma-Aldrich, St. Louis, MO, USA) were filtered using Vivaspin^®^ 500 (Satorius AG, Göttingen, Germany) to eliminate the proteins that could potentially degrade NAD^+^.

### 2.6. Measurement of Cellular Viability and Senescence

HS68 cell viability was measured by the CCK-8 assay according to the manufacturer’s protocol (Cell Counting Kit-8, Dojindo, Kumamoto, Japan). The relative cell population was also measured using this CCK-8 assay.

Cellular senescence was measured based on β-galactosidase activity, which has been widely used in previous studies. For the quantification assay, a plate-based quantification assay kit (Cellular Senescence Plate Assay Kit, SPiDER-βGal, Dojindo, Japan) was used. For imaging senescence, CellEvent™ Senescence Green Detection Kit (Thermo Scientific, San Francisco, CA, USA) was used.

RAW264.7 cell viability was measured using the CCK-8 assay, using 0.1% DMSO in DMEM as a vehicle control blank.

### 2.7. Analysis of Sirtuin and Autophagy Activation

Sirtuin activation was measured by a commercially available assay kit (SIRT1 (Sirtuin1) Fluorogenic Assay Kit, BPS Bioscience, San Diego, CA, USA), with slight modification. A master mix consisting of HDAC, NAD^+^, BSA, and assay buffer was prepared according to the protocol. The cell lysate was prepared using lysis buffer (M-PER™ Mammalian Protein Extraction Reagent, Thermo Scientific, CA, USA). The amount of protein in each lysate sample was measured using BCA assay (protein quantification (BCA), Biomax, Republic of Korea). Cell lysate samples with the same concentration of protein were prepared by diluting cell lysates based on each protein concentration. Cell lysates were mixed with premade master mix and incubated at 37 °C for 30 min. SIRT assay developer was added and the plate was incubated at room temperature for 15 min. The fluorescent intensity (Ex/EM = 360/450 nm) was measured using a microplate spectrophotometer.

Autophagy was analyzed by staining autophagosomes (DAPGreen, Autophagy Detection, Dojindo, Japan). Cells were treated with DAPGreen working solution and then incubated at 37 °C for 30 min. The working solution was removed and the cells were washed with culture medium twice. The medium containing test materials was added to cells for 6 h. After 6 h, the cells were washed with HBSS twice and then the fluorescent intensity (Ex/EM = 488/520 nm) was measured using microplate spectrophotometers. Fluorescent intensities were normalized using the intensities of Hoechst 33342 (Ex/Em = 352/454 nm). For Western blot analysis of LC3B, total protein was extracted in M-PER™ Mammalian Protein Extraction Reagent (Thermo Scientific, CA, USA), and the concentrations of proteins were determined by the BCA assay. The proteins were separated using electrophoresis and transferred onto polyvinylidene difluoride membranes. The membranes were blocked with 5% skim milk and washed with Tris-buffered saline tween (TBST). Afterward, the membranes were incubated with primary antibodies at 4 °C overnight: LC3B (ab192890, 1/1000, Abcam, Cambridge, UK) and β-actin (ab8227, 42 kDa, 1/2000, Abcam Cambridge, UK). After being washed with TBST (3 times/5 min), the membranes were incubated with the secondary antibody horseradish peroxidase-conjugated goat anti-rabbit immunoglobulin G (IgG) H&L (7074S, 1/2000, Cell Signaling Technology, Beverly, MA, USA) for 2 h and then washed with TBST (3 times/50 min) before chemiluminescence development and visualization.

### 2.8. Analysis for DNA Damage and Oxidative Stress

DNA damage was measured using γH2A.X assay and comet assay. Histone H2A.X phosphorylation in response to DNA damage was estimated via the γH2A.X assay, which was performed using a commercially purchased kit from Abcam (Gamma H2A.X Staining Kit, Cambridge, UK). The HS68 cells were seeded on a 12-well cell culture plate one day before the experiment. Prior to UV irradiation, the medium was changed and sample solutions (NAD^+^, Quercetin/Enoxolone complex, NAD^+^/Quercetin/Enoxolone complex) were applied to the cells. After 24 h of incubation, the cells were irradiated with UVB (40 mJ) and re-treated with the sample solution for 24 h. Then, the medium was aspirated off and the cells were washed with DPBS. After fixation using *P*-formaldehyde, ice-cold 90% methanol and blocking solution were added in sequence. Anti-phospho-histone H2A.X antibody solution was added to each well and the cells incubated for 1 h at room temperature. The cells were washed with PBST, a secondary antibody (FITC-conjugated) solution added, and the cells incubated for 1 h at room temperature. Fluorescence images were obtained with fluorescence microscopy using an FITC filter after sufficient washing. For quantification of fluorescence intensity, cells detached using trypsin were fixed and stained as mentioned above. Mean fluorescence intensity per cell was measured using a flow cytometer (CytoFLEX, Beckman Coulter, Brea, CA, USA). Ten thousand cells were analyzed.

In the comet assay, single-cell DNA strand breaks were estimated. It was performed by use of a commercially purchased kit from Abcam (Comet Assay Kit (3-well slides), Cambridge, UK). The HS68 cells were seeded on a 6-well cell culture plate one day before the experiment. Prior to UV irradiation, the medium was changed and the cells were treated with sample solution (NAD^+^, Quercetin/Enoxolone complex, NAD^+^/Quercetin/Enoxolone complex) for 24 h. Then, the cells were irradiated with UVB (40 mJ) and re-treated with the sample solution for 24 h. Cells were collected and suspended in a DPBS/Agarose mix. The agarose/cell mixtures were set on comet slides and treated with lysis buffer (45 min) and alkaline electrophoresis solution (30 min), following the manufacturer’s protocol. Electrophoresis was performed under alkaline conditions, with 1 V/cm for 15 min. After electrophoresis, the slides were washed with water and pre-chilled 70% Ethanol. After drying, the slides were stained with Vista Green DNA Dye for 15 min. Fluorescence images were obtained with fluorescence microscopy using an FITC filter. The Tail DNA intensity and the tail moment length, which both reflect DNA damage in host cells, were analyzed by CapsLab software (1.2.3b1). Ten cells in the same agarose spot in the same glass slide were randomly chosen and analyzed. Results are presented as Tail DNA (%) and Olive Tail Moment using the following formula: Tail DNA (%) = Tail DNA intensity/Cell DNA intensity × 100; Olive Tail Moment = Tail DNA (%)/Tail moment length.

Cellular oxidative stress was estimated using the DCFDA assay. It was performed using a commercially purchased kit from Abcam (DCFDA/H2DCFDA, Cellular ROS Assay Kit, Cambridge, UK). The HS68 cells were seeded on a black flat clear bottom 96-well cell culture plate at a density of 2 × 10^4^ cells/well in 100 μL of culture medium and incubated for 24 h. After replacing the medium with fresh medium, the cells were treated with test materials. After 24 h of incubation, DCFDA solution (20 μM in fresh medium) was added and the cells treated for 3 h to stain cellular reactive oxygen species. Then, the medium was aspirated off and H_2_O_2_ solution (500 μM) with the compound of interest (NAD^+^, Quercetin/Enoxolone complex, NAD^+^/Quercetin/Enoxolone complex) was added. After 45 min, fluorescence was measured at Ex/Em = 485/535 nm in the presence of the compound or medium.

### 2.9. Wound Scratch Assay

Fibroblasts were seeded in 24-well plates. One day after, 10 μM of FK866 (Daporinad, noncompetitive inhibitor of nicotinamide phosphoribosyl transferase (NAMPT), Cas 658084-64-1) was applied and cells were cultured for an additional 15 h. Culture supernatants were removed and scratches were uniformly generated using a scratcher (SPLScar^TM^ Scartcher, SPL, Pocheon, Republic of Korea). Cells were washed with PBS once and supplemented with culture media containing test materials. The wound areas were analyzed using microscopy after 15 h.

### 2.10. Analysis of Mitochondrial Potential and Oxidative Stress

As described in previous studies, the mitochondrial membrane potentials and their kinetics were analyzed using TMRM (tetramethylrhodamine, methyl ester) [32,33]. The mitochondrial uncoupler FCCP (Carbonyl cyanide *P*-trifluoro methoxyphenylhydrazone) was used for membrane potential eradication. Cells were seeded in 96-well black plates, and one day after, FK866 was applied for 24 h. Then, test materials for NAD^+^, NAD^+^, and boosting complex were added to the cells, and the cells were incubated for a further 48 h. For treatment of FCCP to establish the intrinsic baseline control, 20 μM of FCCP was added to cells in serum-free medium 15 min prior to TMRM staining. TMRM (20 μM) in HBSS was applied to cells for 45 min and the cells washed twice with HBSS. The nucleus was stained with Hoechst 33342. The fluorescent intensities (Ex/Em = 548/573 for TMRM, Ex/Em = 352/454 for Hoechst 33342) were measured by spectroscopy using the well-reading mode. TMRM intensities were normalized by dividing by nucleus intensities. For microscopic analysis of kinetics, only bright spots or areas with TMRM staining were focused on and FCCP was added. The videos were obtained and analyzed using Image J (1.52o version), as described in the previous study [34].

For analysis of mitochondrial mass and populations and oxidative stress, MitoTracker^TM^ and MiSox^TM^ were used according to the manufacturers’ protocols. Mean fluorescence intensities per cell for MitoTracker^TM^ and MitoSox^TM^ were analyzed by a cytometer (CytoFLEX, Beckman Coulter, Brea, CA, USA). Ten thousand cells were analyzed. MitoTracker^TM^ was used for analyzing mitochondrial mass and MitoSox^TM^ was used for analyzing mitochondrial oxidative stress [35].

### 2.11. Anti-Inflammatory Activity of NAD^+^ and Boosting Complex

Anti-inflammatory activity was measured through inhibition of LPS-induced NO production in RAW264.7 cells by the Griess method [36]. This experiment included a vehicle group (0.1% DMSO), a model group (LPS), a positive control group (aminoguanidine hydrochloride), and a sample-treated group (NAD^+^ and boosting complex).

The inhibition of some pro-inflammatory cytokines (TNF-α, IL-1β, and IL-6) by NAD^+^ and boosting complex was also estimated in LPS-induced RAW264.7 cells using commercial ELISA kits (Shanghai Weiao Biotechnology Co., Ltd., Shanghai, China).

### 2.12. Statistical Analysis

Data are presented as mean values ± SDs derived from at least three independent experiments. Statistical analyses of data were performed using Student’s *t*-test and one-way ANOVA (Dunnett’s test). Dunnett’s test was performed to analyze the significance between the control group and experimental groups. A *p*-value less than 0.05 (* *p* < 0.05 or # *p* < 0.05) indicated a significant difference.

## 3. Results and Discussion

### 3.1. Inhibition of CD38 Expression and the Elevation of Cellular NAD by Quercetin and Enoxlone Under Exogenous NAD^+^ Supplementation

Despite different methodologies and argumentation for discrimination of free and protein-bound NAD^+^, the intracellular NAD^+^ level is estimated to be between 0.2 and 0.5 mM (132.69–331.7 ppm) [37]. Although it is debatable whether NAD^+^ and NAD^+^ precursors can permeate the cell membrane, some studies have shown that topical supplementation of exogenous NAD^+^ can elevate both cellular NAD^+^ and total systematic NAD^+^ levels. In neurons, NAD^+^ is taken up by cells through connexin 43 (CX43) [38]. In zebrafish larvae, exogenous NAD^+^ (~1 mM) added to larval water significantly increased larval NAD^+^ levels [39,40]. Given this experimental evidence, NAD^+^ itself may be delivered into cells, although the transport system for exogenous NAD^+^ is not clearly characterized at present. The discovery of the elusive NMN transporter, Slc12a8, in 2019, during a furious debate on whether NMN is cell permeable, is noteworthy [41].

**Figure 1:**
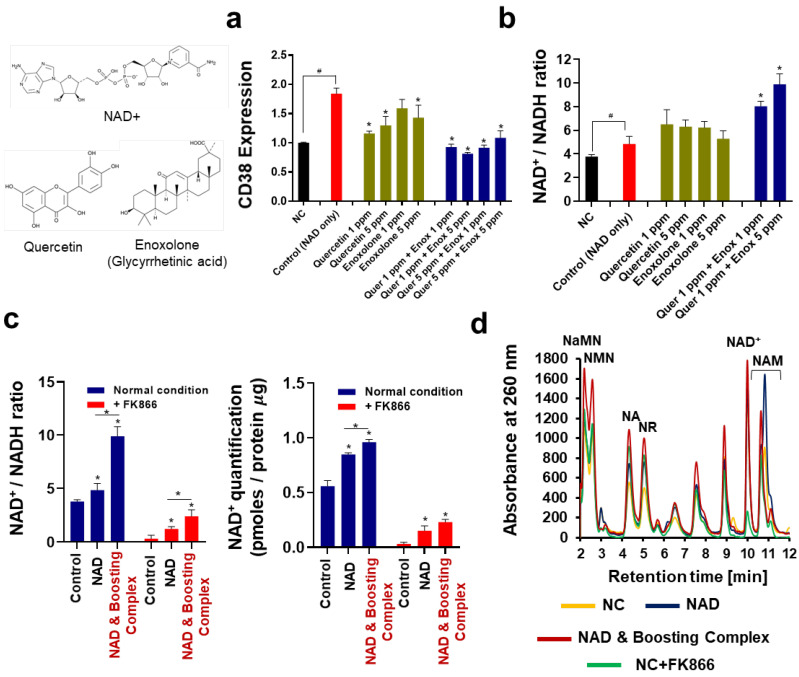
Cellular NAD^+^ elevation by CD38 inhibition under supplementation of exogenous NAD^+^. (**a**) CD38 expression analyzed by real-time qualitative polymerase chain reaction (RT-qPCR). All groups were treated with NAD^+^ (20 ppm), apart from the NC. “Quer” refers to quercetin and “Enox” refers to enoxolone. (**b**) NAD^+^/NADH ratio. Each mole of NAD^+^ and NADH was determined and then calculated into a ratio. (**c**) Quantification of cellular NAD^+^ under NAD-depleted conditions. An FK866 (10 μM) treatment was applied. NAD and Boosting complex refers to the co-treatment of NAD^+^, quercetin (1 ppm), and enoxolone (5 ppm). (**d**) NAD^+^-related metabolite analysis using high-performance liquid chromatography (HPLC). All experiments were performed in triplicate, except the quantification of NAD^+^. Experiments for quantification of NAD^+^ (Figure 1c) were performed in quadruplicate. Student’s *t*-test was performed to show the difference between NC and control group (# Significantly different results (*p* < 0.05)). One-way ANOVA (Dunnett’s test) was performed for comparison between control and experimental groups (* Significantly different results (*p* < 0.05)).

When exogenous NAD^+^ was added to the cells, a 1.84-fold overexpression of CD38 was observed (Figure 1a). CD38, also known as cyclic ADP ribose hydrolase, is a multifunctional enzyme that uses NAD^+^ as a substrate to generate secondary messengers. CD38 targets the N-ribosyl bond of NAD^+^ molecules and converts NAD^+^ to NAM and ADP-ribose, which are subsequently cyclized to cADP-ribose [42]. Recent studies have shown that CD38 plays a critical role in regulating cellular NAD^+^ levels and is highly correlated with NAD^+^ decline under both pathological conditions and intrinsic aging [43]. Numerous studies have verified that CD38 overexpression results in a decrease in NAD^+^, and downregulation of CD38 inhibition results in an increase in NAD^+^ in cells. While the detrimental effects of CD38 on NAD^+^ level are debated, recent research also indicates the role of CD38 in NAD homeostasis as an “arbitrator” under normal cell conditions [42]. Our observation suggests that overexpression of CD38 by exogenous NAD^+^ may be a homeostatic mechanism to counteract excessive cellular NAD^+^. Overexpression of CD38 by exogenous NAD^+^ was effectively inhibited by quercetin and enoxolone, and this inhibitory effect was synergistically maximized when they were co-administered. The maximum inhibitory effect on CD38 expression, 55.82% inhibition efficiency (0.813), was observed with 1 ppm of quercetin and 5 ppm of enoxolone. We investigated whether this quercetin–enoxolone complex elevates cellular NAD^+^ levels when supplemented with exogenous NAD^+^ (Figure 1b). Exogenous NAD^+^ at 20 ppm increased the cellular NAD^+^/NADH ratio from 3.79 to 4.84, a 1.28-fold increase. Quercetin and enoxolone increased the NAD^+^/NADH ratio. Consistent with the previous experiment, co-treatment with quercetin and enoxolone dramatically increased the cellular NAD^+^/NADH ratio to 9.91, which was 2.04-fold higher than that of the control. In the NAD^+^-depleted condition induced by FK866, co-treatment with 20 ppm NAD^+^, 1 ppm quercetin, and 5 ppm enoxolone (hereafter, the combination of 1 ppm quercetin and 5 ppm enoxolone is referred to as the boosting complex) also significantly increased NAD^+^ levels (Figure 1c). It was observed that both the NAD^+^/NADH ratio and the intracellular NAD^+^ concentration significantly increased when NAD^+^ and boosting complex were added. In the quantification of the intracellular NAD^+^ concentration, the differences between the NAD^+^-only group and the NAD and boosting complex were smaller than that of NAD^+^/NADH ratio data.

Next, we analyzed NAD^+^ metabolites using HPLC (Figure 1d). The dominant peak of NAM was observed when exogenous NAD^+^ was supplemented (Dark blue), which seems to be mediated by CD38-mediated salvage. Interestingly, in the spectrum of the NAD^+^–booster complex-treated group (Red), the NAM peak was shown to be slightly decreased, which might be due to the suppressed CD38-mediated salvage. Further in-depth investigation of NAD^+^ metabolites will offer clearer clues as to how exogenous NAD^+^ is transported and metabolized.

Thus, it can be concluded that the boosting complex effectively enhances the bioavailability of exogenous NAD^+^.

### 3.2. Protective Effects of theNAD^+^–Boosting Complex on UV-Induced Photoaging

UV light is considered the most critical factor in skin aging. A pioneering study that demonstrated molecular actions in UV-irradiated skin [28] established various approaches for protecting skin against UV-induced photoaging. Interestingly, recent research has also verified that UVB irradiation decreases cellular NAD^+^ and consequently induces a drastic PARP-dependent loss of cellular metabolic activity [44]. Later studies revealed that restoring NAD^+^ by overexpressing NAMPT [45] or supplementing the NAD^+^ precursor through intraperitoneal injection could relieve the adverse impact of UV on skin cells [46].

**Figure 2:**
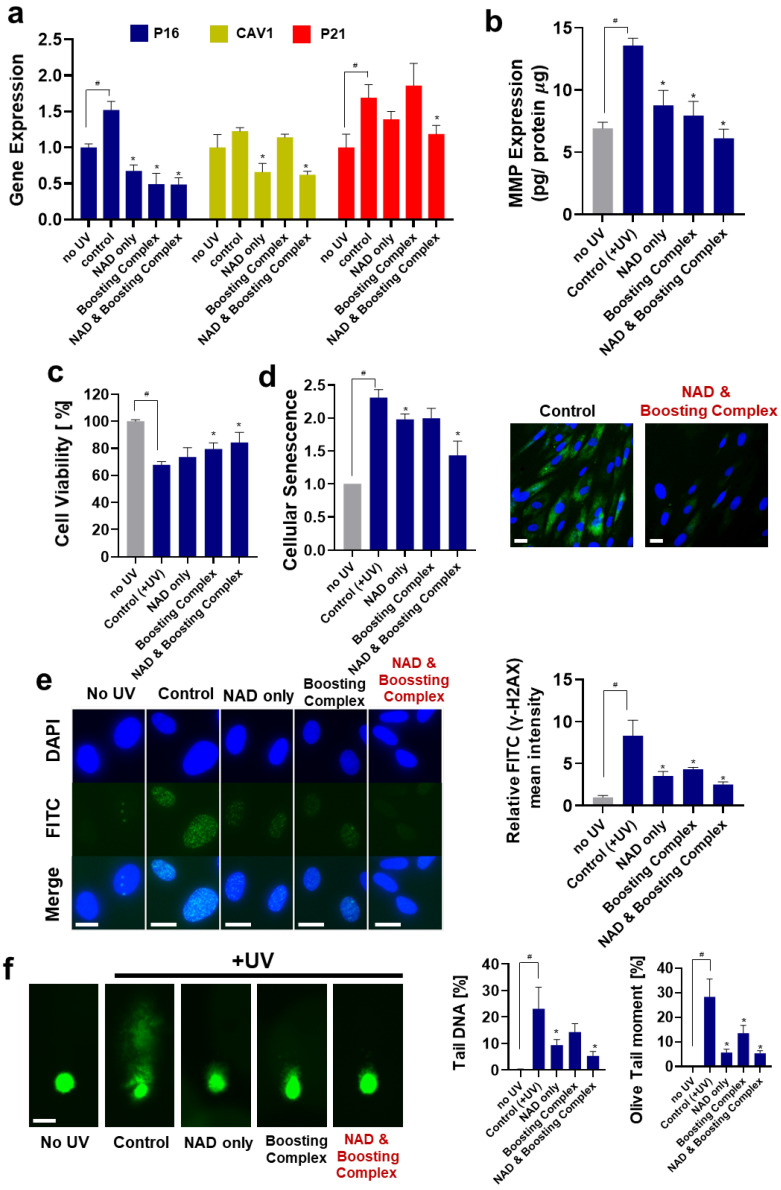
Protective effects of exogenous NAD^+^ on UV-induced photoaging. (**a**) Expression of aging-related genes analyzed by RT-qPCR. All groups except “no UV” were treated with UV irradiation. Boosting complex refers to the combination of quercetin (1 ppm) and enoxolone (5 ppm). The NAD^+^ treatment concentration was 20 ppm. (**b**) MMP-1 expression was analyzed by ELISA. The amount of MMP-1 protein was quantified and normalized using the amounts of protein. (**c**) Photo-toxicity data. Cell viabilities were measured using cell counting kit-8 (CCK-8). (**d**) Cellular Senescence data. β-galactosidase activities in cell lysates were measured and normalized by protein amounts. For image analysis, cells were stained after fixation. Scale bar (White) = 10 μm. (**e**) γ-H2AX for analysis of DNA damage under UV irradiation. Mean fluorescence intensity per cell was measured using a flow cytometer and normalized. Scale bar (White) = 10 μm. (**f**) Comet assay for analysis of genomic DNA integrity and damage under UV irradiation. Scale bar (White) = 20 μm. All experiments were performed in triplicate except for the senescence experiment (performed in quadruplicate). Student’s *t*-test was performed to determine the significance between negative control (no UV) and control groups (# Significantly different results (*p* < 0.05)). One-way ANOVA (Dunnett’s test) was performed for comparison between control and experimental groups (* Significantly different results (*p* < 0.05)).

Here, we investigated whether elevating cellular NAD^+^ with the boosting complex could protect against UV-induced photoaging at the in vitro level (Figure 2a). As mentioned in previous studies [47,48], P16, CAV1, and P21, which reflect the aging progress, were overexpressed by 52.13%, 22.52%, and 68.96%, respectively. Treatment with NAD^+^ and the boosting complex effectively decreased the UV-induced overexpression of these three genes. This combination was more potent than NAD^+^ alone, exhibiting decreases of 68.05%, 49.26%, and 29.73% in P16, CAV1, and P21 expression, respectively. Interestingly, treatment with the boosting complex without NAD^+^ effectively suppressed UV-induced overexpression of P16, while this effect was not observed in other genes.

MMP overexpression by UV irradiation is a major trigger of wrinkle formation in the skin [49]. Numerous strategies, such as inhibition of MMP expression or neutralization of enzymatic activity, have been suggested. We tested the effect of NAD^+^ and the NAD^+^-boosting complex on the UV-induced overexpression of MMP-1 (Figure 2b). The 1.96-fold increase in MMP-1 decreased when treated with both NAD^+^ and the boosting complex. The NAD^+^ and boosting complex was most effective in suppressing UV-induced MMP-1 overexpression, showing a 54.95% decrease compared with the control group, although statistical significance was not observed between these two groups (*p*-value = 0.0532).

Cell viability and senescence were analyzed. We observed that both NAD^+^ and boosting complex could effectively reduce UV-mediated cytotoxicity, and the combination of NAD^+^ and boosting complex was the most potent, with a 24.40% increase in viability compared to the control. In the analysis of cellular senescence, the UV-induced 2.31-fold increase in cellular senescence was dramatically decreased by 37.88% (1.435) when NAD^+^ and boosting complex was used.

To investigate the effect of NAD^+^ and boosting complex on UV-induced DNA damage, we performed a γ-H2AX assay, which detects γ-H2AX, a sensitive molecular marker of double-stranded DNA damage [50], as well as a comet assay. As shown in Figure 2e, UV irradiation caused significant DNA damage, which was effectively alleviated by NAD^+^ and NAD^+^ and boosting complex. In the comet assay, the longer fluorescent tail representing DNA fragmented by UV irradiation dramatically decreased when treated with NAD^+^, boosting complex, and NAD^+^ and boosting complex (Figure 2f). Previous studies demonstrated that NAD^+^ is consumed in response to DNA damage, and highly contributes to DNA repair in a PARP-dependent manner [51]. Our experiment results regarding the alleviation of DNA damage by elevation of cellular NAD^+^ are consistent with these previous studies [51]. It can be concluded that elevated cellular NAD^+^ has a protective role against UV-mediated DNA damage and enables higher cellular viability and the inhibition of cellular senescence. Similar to the UV-induced aging model, we investigated the protective effects of NAD^+^ on the H_2_O_2_-induced oxidative stress model. As shown in Figure S1, the elevated oxidative stress was suppressed when NAD^+^ or NAD^+^ and boosting complex were added, showing decreases of 13.71% and 20.57%, respectively.

### 3.3. NAD^+^ and Boosting Complex Effectively Alleviate Intrinsic Aging and Extend Replicative Lifespan of Fibroblasts

Replicative senescence refers to the phenomenon whereby normal non-malignant cells stop dividing after reaching a limited number of replications, termed the Hayflick limit [52]. With each round of DNA replication, telomeres progressively shorten and eventually reach a critical length that prevents further replication, thereby halting cell division [53]. The combination of telomere shortening and accumulated DNA damage results in a limited replicative lifespan, which is regarded as the most critical triggering point for intrinsic aging at both in vitro and in vivo levels [54,55]. The relationship between cellular aging in vitro and actual in vivo skin aging phenotypes, such as wrinkles, has been discussed in the past decades [56,57].

Here, we investigated how NAD^+^ and NAD^+^ and boosting complex ameliorate intrinsic aging. Firstly, sirtuin activation was measured. Sirtuins, also sometimes called the “longevity genes” (or “longevity proteins”) have been intensively studied and become a hot topic in aging science because of increasing evidence of the role of sirtuins in aging and age-related diseases [58]. While their mechanism is not fully understood, they play roles in preventing telomere attrition, promoting DNA damage repair, and sustaining genome integrity [59]. Owing to their extensive protective actions against aging, their effects on lifespan extension have been demonstrated in various experimental models, including in yeast [60], *C. elegans* [61], *Drosophila* [62], and mice [63]. In a skin model, activation of sirtuin activity or elevated SIRT1 expression has been shown to be effective against oxidative stress and UV-induced aging [64,65]. In addition, disrupted epidermal barrier function due to decreased filaggrin expression in aged skin may be related to a decline in SIRT1 activation [66,67].

**Figure 3:**
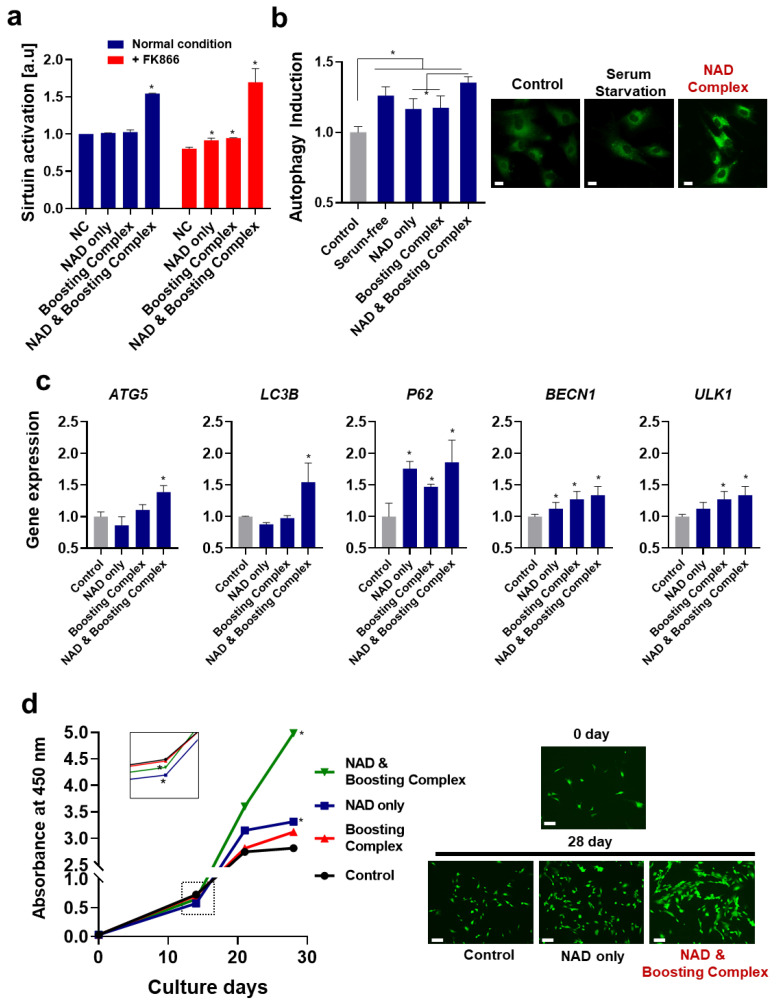
Elevation of NAD^+^ enhances sirtuin activation and autophagy induction and extends the replicative lifespan. (**a**) Sirtuin activation of cell lysates was analyzed. (**b**) Autophagy induction: the degree of autophagy was measured using an autophagosome-staining dye. Scale bar (White) = 10 μm. (**c**) RT-qPCR was performed to analyze autophagy-related gene expression. (**d**) Replicative lifespan of fibroblasts: cells were cultured for 4 weeks, and cell populations were measured using CCK-8. The absorbance at 450 nm proportional to the number of cells was measured. Visualization was achieved by calcein-AM staining. Scale bar (White) = 20 μm. All experiments were performed in triplicate. One-way ANOVA (Dunnett’s test) was performed for comparison between control and experimental groups. * Significantly different results (*p* < 0.05).

In our experiments, significantly increased sirtuin activation was observed when fibroblasts were treated with NAD^+^ and booster complex (Figure 3a), and this effect was more prominent under NAD^+^ depletion conditions (FK866). The treatment with quercetin and enoxolone was not as effective as treatment with NAD^+^ and boosting complex, although quercetin is also known as a sirtuin activator [68]. This marginal effect seems to be due to insufficient intracellular concentrations, while the IC_50_ of quercetin for activating SIRT6 is 1.2 mM (362.4 ppm) [69]; additionally, it may also be due to the inhibitory effects of quercetin on other sirtuins (Sirtuins 1, 2, 3, and 5) at low concentrations [69]. This observation proves that sirtuin activation is not mediated by the phytochemicals themselves but by the elevation of cellular NAD^+^.

Therefore, we investigated the induction of autophagy. As shown in Figure 3b, all groups showed enhanced autophagy, and NAD^+^ and booster complex synergistically maximized this effect, showing a 1.35-fold increase in autophagy. The expression levels of autophagy-related genes (*ATG5*, *LC3B*, *P62*, *BECN1*, and *ULK*) were analyzed (Figure 3c). As in previous experimental results, NAD^+^ and the boosting complex induced the highest expression of each gene, with increases of 1.39-fold for *ATG5*, 1.54-fold for *LC3B*, 1.86-fold for *P62*, 1.34-fold for *BECN1*, and 1.29-fold for *ULK1*. When protein expressions of LC3B (LC3B-I and LC3B-II) were analyzed using Western blot, the enhanced expression of LC3B-II was not observed. In contrast, a slight increase in LC3B-I expression was observed, presumably suggesting that other autophagy factors crucially exert autophagy induction. (Supplementary Figure S2). It is noteworthy that some autophagy cargo fluxes are driven in an LC3B-independent manner [70]. The bidirectional relationship between NAD^+^ and autophagy has been well demonstrated in previous studies; NAD^+^ is an autophagy regulator, while autophagy also controls cellular NAD^+^ levels. Our observation is consistent with previous findings that elevation of cellular NAD^+^ enhances autophagy [71,72,73]. In addition, autophagy induction by NAD^+^ seems to be highly associated with sirtuin activation, as described in previous studies demonstrating that sirtuin activation induces autophagy via various pathways such as FOXO1 1/Rab7 or Bnip/NIX pathways [74]. This sirtuin-activation-induced autophagy also critically contributes to delayed aging and life extension. The previous observation that the lifespan-extending effect in *C. elegans* by overexpression of sirtuin was compromised when Bec-1, one of the key proteins of autophagy, was knocked out supports this idea [75].

We analyzed the replicative reproduction ability of fibroblasts, that is, how cells can replicate during long-term cultivation, for 4 weeks. As shown in Figure 3d, the control (untreated), NAD^+^-only, and boosting complex groups showed dramatically decreased cell division after 3 weeks and reached a plateau. Meanwhile, the NAD^+^ and boosting complex group showed a continuous increase in cell population. After 4 weeks, as an arbitrary value, the cell populations of the NAD^+^ and boosting complex, NAD-only, boosting complex, and control groups were 4.99, 3.12, 3.31, and 2.81, respectively. Of note, NAD^+^-only and NAD and boosting complex groups exhibited slightly lower cell populations at 2 weeks, which was not only statistically significant, but also observed in repeated experiments (dotted box in the graph). One possible explanation for this is enhanced autophagy. Although the question regarding the role of autophagy in the cell cycle is controversial [76], it has long been argued that autophagy may inhibit cell division and proliferation under most conditions. However, there are contradictory findings on whether autophagy promotes wound healing. The fact that rapamycin treatment was shown to cause delayed wound closure in mice at early time points before showing similar wound closure outcomes at later times deserves attention [77]. Furthermore, this occurs despite the common notion that autophagy plays an important role in chronic wounds and dermal proliferation [78]. Taken together, these previous observations and our experimental results support the idea that, although autophagy inhibits cell proliferation in the early stages to renovate cellular organelles and proteostasis, it consequently improves cellular functions, later enhancing replication and longevity. This is supported by numerous studies that have shown that increased autophagy is associated with delayed aging or life extension [79]. Therefore, the higher cell populations and extended replicative lifespan could be partially explained by enhanced autophagy induced by elevated cellular NAD^+^ levels.

### 3.4. Reversal of Suppressed Wound Healing and Proliferation Effect of NAD^+^ and Boosting Complex under NAD^+^ Depletion

**Figure 4:**
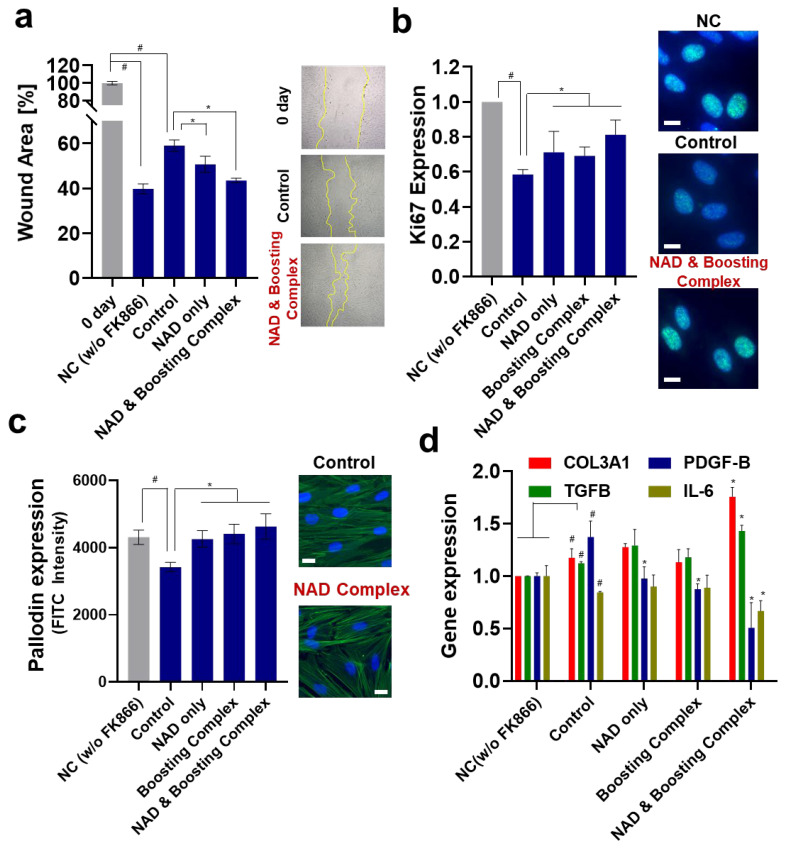
Elevation of NAD^+^ enhances wound healing of fibroblasts. (**a**) Wound scratch assay: the wound area was measured after 24 h. (**b**) Ki67 expression: the FITC field intensity (ex/em = 488/520) was measured and normalized against the nuclear staining intensity (Hoechst 33342). Scale bar (White) = 20 μm. (**c**) Actin/phallodin staining: similar to Ki67, FITC intensity was measured and normalized using nuclear staining intensity (Hoechst 33342). Scale bar (White) = 20 μm. (**d**) RT-qPCR was performed to assess wound-healing-related gene expression. All experiments were performed in triplicate. Student’s *t*-test was performed to determine the significance between negative control and control group (# Significantly different results (*p* < 0.05)). One-way ANOVA (Dunnett’s test) was performed for comparison between control and experimental groups (* Significantly different results (*p* < 0.05)).

A growing body of evidence suggests that NAD^+^ homeostasis is critical for both cell proliferation and wound healing; further, elevation of NAD^+^ could promote wound healing in the skin [80]. This effect is presumed to occur through the Sirt1/AMPK pathway or via Pgc-1α. Although numerous mechanisms for delayed wound healing in aged skin are known, the age-related decline in NAD^+^ may also contribute to impaired skin wound healing. Here, we investigated the effects of an NAD^+^-boosting complex on wound healing using a fibroblast-mediated scratch assay (Figure 4a and Figure S3).

Similar to previous studies that observed that inhibiting NAMPT by FK866 exhibited decreased cell proliferation for diverse cell lines [81,82] and that NAD^+^-depleted condition due to hyperglycemia triggered impaired corneal epithelial wound healing [80], FK866 treatment significantly inhibited wound closure. Treatment with NAD^+^ alone or NAD^+^ and boosting complex effectively decreased the wound area to a greater extent than the control, showing 50.76% and 43.58% reductions in wound areas, respectively. Ki67, a cellular proliferation marker, was also analyzed (Figure 4b). As in the scratch assay, FK866 treatment dramatically decreased the ki67 expression by about 41.6%. NAD^+^ and NAD^+^ and booster complexes restored the decreased ki67 expression to 0.712 and 0.812, which was an 18.41% and 39.04% increase compared to the control group. When F-actin levels were analyzed, a similar trend was observed, showing the highest FITC intensity in the NAD^+^ and booster complex group, although its difference compared to the NAD^+^-only group was not significant (Figure 4c). When mRNA expressions of ACTG1 (Monomeric G actin), ARP2 (Actin-related protein 2), and ARP3 (Actin-related protein 3) were additionally analyzed (Supplementary Figure S4), it was observed that FK866 decreased the expressions of ACTG1 and ARP2, and NAD^+^ and booster complex restored decreased expression. Although FK 866 did not affect the expression level of ARP3, boosting complex and NAD^+^ and boosting complex increased ARP3 expression. Our observation that better wound healing or increased cell proliferation are accompanied by higher expression of cell cytoskeleton proteins, such as actin, is consistent with previous studies [83,84,85]. The expression of genes considered to be related to wound healing was investigated (Figure 4c). Interestingly, NAD depletion upregulated COL3A1, PDGF-B, and TGFB expression, whereas that of IL-6 was downregulated. Although they are well-known wound healing-related genes, we can only make limited inferences due to the absence of studies investigating their relationships with cellular NAD^+^ levels. Previous studies observed that *NAMPT* knockout increased the TGF-b1 level [86], while FK866 treatment of pulmonary fibrotic tissue upregulated COL3A1 while downregulating IL-6 [87]. Of note, NAD^+^ depletion by FK866 induces autophagy and PDGF also induces autophagy [88]; thus, it is likely that FK866-induced autophagy could be the result of PDGF upregulation. NAD^+^ treatment decreased its expression, which could be regarded as autophagy homeostasis against exogenous NAD^+^, as previously described [89,90].

When the cells were treated with the NAD^+^-boosting complex, COL3A1 and TGFB were upregulated, whereas PDGF-B and IL-6 were downregulated. The decreased expression of IL-6 seen here is similar to that in previous studies that observed that the administration of NAD^+^ precursors suppresses IL-6 expression in mice and humans [91,92].

Taken together, NAD^+^-depletion-induced suppression of wound healing was effectively rescued through the elevation of cellular NAD^+^ levels, and this was supported by the elevated expression of proliferation markers such as Ki67, COL3A1, and TGFB. For an in-depth understanding of how NAD^+^ governs the progress of cellular proliferation in wound healing, a broader investigation based on whole-genome transcriptome analysis or proteomics analysis is required.

### 3.5. NAD^+^ and Boosting Complex Restores Mitochondrial Functionality under NAD^+^ Depletion

**Figure 5:**
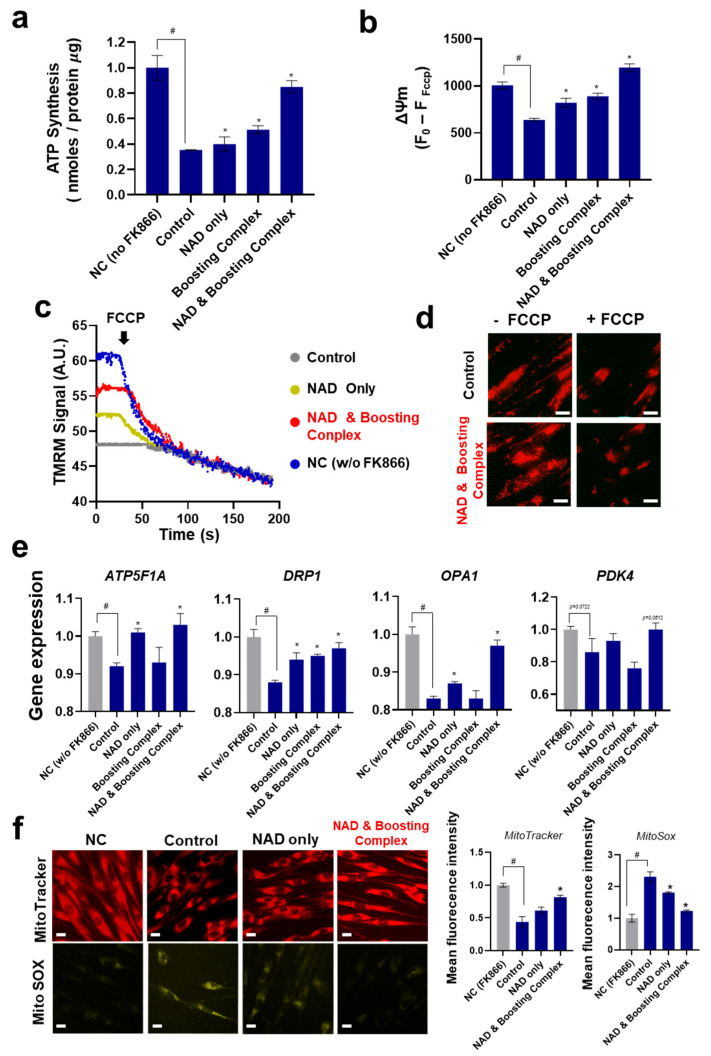
Elevation of NAD^+^ mitochondrial functionalities. (**a**) ATP synthesis normalized by protein amount of cell lysates. (**b**) Mitochondrial membrane potentials (ΔΨm). Tetramethylrhodamine methyl ester (TMRM) intensities were measured. (**c**) Kinetics for ΔΨm. (**d**) TMRM staining under fluorescent microscopy. Scale bar (White) = 10 μm. (**e**) The expression levels of genes related to mitochondrial functionalities and morphology were assessed by RT-qPCR. (**f**) Microscopic analysis for visualization of mitochondria and ROS. Scale bar (White) = 10 μm. Fluorescence intensities for MitoTracker and MitoSox were analyzed by flow cytometry. Mean fluorescence intensity per cell was analyzed. All experiments were performed in triplicate. Student’s *t*-test was performed to determine the significance between negative control and control group (# Significantly different results (*p* < 0.05)). One-way ANOVA (Dunnett’s test) was performed for comparison between control and experimental groups (* Significantly different results (*p* < 0.05)).

We investigated how FK866-induced NAD^+^ depletion affects the mitochondrial function of fibroblasts and how it can be restored using exogenous NAD^+^. ATP synthesis, a major mitochondrial function, was analyzed (Figure 5a). As expected, FK866-induced NAD^+^ depletion dramatically decreased ATP synthesis by approximately 64.7%, in line with previous reports [93]. This inhibition of ATP synthesis was slightly rescued when NAD^+^ and boosting complex were added, but the difference was not statistically significant. NAD^+^ and boosting complex effectively enhanced ATP synthesis by 140.56% compared with the control.

ΔΨm was analyzed (Figure 5b,c). ΔΨm, defined as the difference in electrical potential between the mitochondrial matrix and the mitochondrial transmembrane space, is generated by proton pumps (complexes I, III, and IV); thus, it is also regarded as a key indicator of mitochondrial activity [94,95,96]. Similar to ATP synthesis, the mitochondrial potential was significantly decreased by 36.43% with FK866 treatment, while treatment with NAD^+^ and boosting complex dramatically increased the mitochondrial potential by 86.91% compared to the control. TMRM staining, which visualizes the membrane-potential-active mitochondria, revealed stronger fluorescent signals in the NAD^+^–boosting complex-treated group (Figure 5d).

Genes related to ATP synthesis and mitochondrial homeostasis, including mitochondrial fusion and fission, were analyzed. Treatment of HS68 with FK866 downregulated the expression levels of *ATP5F1A* (8%), *DRP1* (12.15%), and *OPA1* (17.12%) (Figure 5e). These suppressed gene expressions were recovered when NAD^+^ or NAD^+^ and boosting complex were added, with the combined treatment being the most potent enhancer of the expression of these genes.

The change in *ATP5F1A* expression supports previous observations that NAD^+^ depletion induces a decrease in ATP synthesis and that this effect is compromised by the elevation of cellular NAD^+^ [97].

The variation in *PDK4* expression was less significant than that of the other genes. PDK4 controls the carbon flux into glycolysis by inhibiting the pyruvate dehydrogenase complex [98]. Even though recent research argues that PDK4 mediates energy stress-induced mitochondrial fission and acts in metabolic adaptation against metabolic stress [96], significant variations in the expression of PDK4 under NAD^+^-depleted conditions and under NAD^+^ supplementation were not observed in our experiment. Although *DRP1* and *OPA1*, the representative genes for mitochondrial fission and fusion, respectively, were downregulated and rescued by the NAD^+^ and boosting complex, no clear differences in mitochondrial homeostasis between fusion and fission were detected in our microscopic observation.

Mitochondrial analysis was performed using MitoTracker and MitoSox. Mitochondria were stained with MitoTracker to determine mitochondrial mass and with MitoSox to detect mitochondrial reactive oxygen species. Similar to previous experimental results of TMRM staining, we observed decreased mitochondrial mass induced by NAD^+^ depletion and its recovery by NAD^+^ or NAD^+^ and boosting complex. In the case of MitoSOX, we observed that NAD^+^ depletion led to mitochondrial oxidative stress, as previously reported [99]. Mitochondrial oxidative stress is mitigated by elevated cellular NAD^+^ levels. Considering that mitochondrial reactive oxygen species are closely related to cellular senescence, inhibiting mitochondrial reactive oxygen species generation could contribute to the mitigation of UV-induced senescence.

Considering all the experimental results obtained above, it can be concluded that NAD^+^ depletion induces adverse effects on the mitochondrial functions of fibroblasts and that these effects can be effectively mitigated by elevating cellular NAD^+^ levels, as they respond similarly to other cell lines. To the best of our knowledge, this is the first observation of this phenomenon in fibroblasts.

There are several limitations that complicate in-depth interpretation. First, the intimate crosstalk between cytosolic or nuclear events with mitochondria remains unclear. Second, there are still uncertainties regarding mitochondrial dynamics, such as whether mitochondrial fusion or fission is more dominant with NAD^+^ supplementation. These questions appear to be intractable, as numerous studies on mitochondrial function and homeostasis have shown inconsistent data and conclusions [100,101,102]. More extensive and ultramicroscopic research on mitochondrial morphology and functionality, which might offer clues to answer these questions, will be necessary in the future.

### 3.6. Anti-Inflammatory Activity of NAD^+^ and Boosting Complex against NO Production and Pro-Inflammatory Cytokines

Skin as a vast organ with immunological function governs numerous important inflammatory and immunological processes against continual interaction with external environments [103]. Inflammation of the skin, whether it is intense or mild, is quite ubiquitous in human life, and overreacting inflammatory reactions in the skin can cause not only severe diseases such as dermatitis but also sensitive skin syndrome and erythema in daily life [104]. It is considered that anti-inflammation of the skin could result in numerous benefits, hence diverse approaches have been investigated [105].

Recent studies have revealed that chronic physiological stimulation of the immune system or inflammation over a long time results in the accumulation of damage to tissues and organs, which finally leads to the development of aging [106]. This phenomenon on the skin, also called inflammaging, has also been investigated by showing that senescent skin cells, such as keratinocytes, fibroblast, and even numerous immune cells, secrete substantial inflammatory cytokines and, following heightened immune system responses, potentially contribute to inflammaging and abnormal autoimmunity [107]. In this section, we investigated whether NAD^+^ and two phytochemicals, quercetin and enoxolone, have anti-inflammatory effects and could potentially contribute to ameliorating inflammaging. To mimic the hypersensitive immune system, the LPS-induced RAW264.7 cell model was used. RAW264.7, one of the monocyte/macrophage-like cell lines, has been utilized for studying immune responses in skin [108,109] and macrophage senescence and NLRP3-mediated inflammaging [110,111,112].

**Figure 6:**
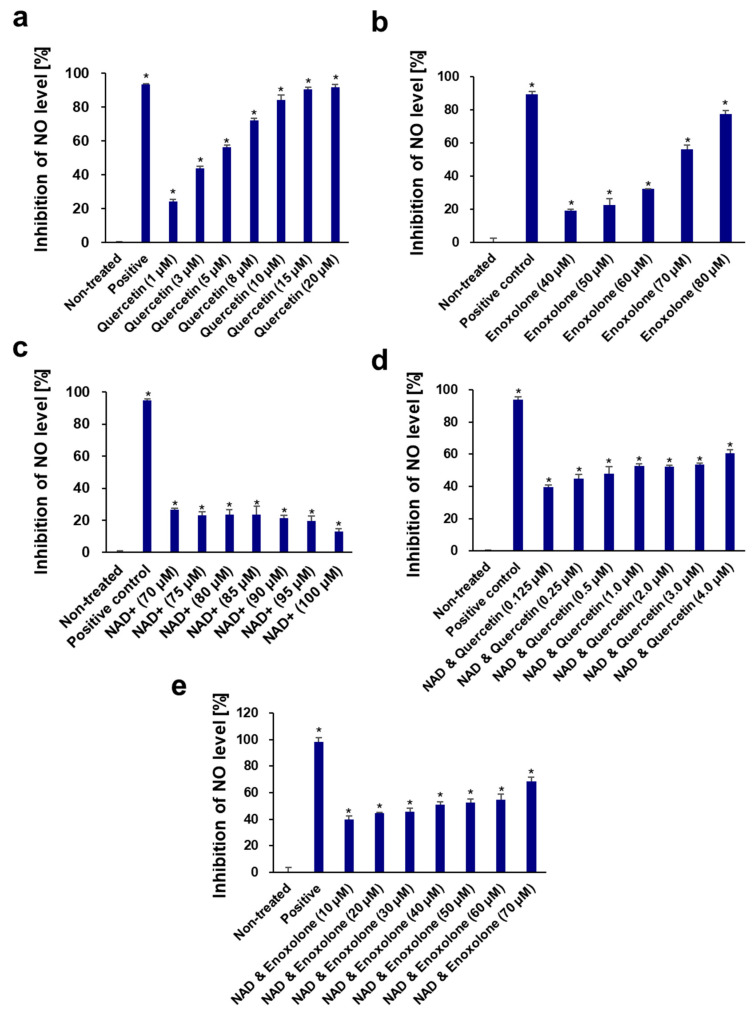
Dose-dependent effects of quercetin (**a**), enoxolone (**b**), NAD^+^ (**c**), NAD^+^ and quercetin (**d**), and NAD^+^ and enoxolone (**e**) on inhibition of NO levels in LPS-induced RAW264.7 cells. Aminoguanidine hydrochloride was used as the positive control. All experiments were performed in triplicate. Student’s *t*-test was performed to determine significance between the non-treated group and test groups (* Significantly different results (*p* < 0.05)).

**Figure 7:**
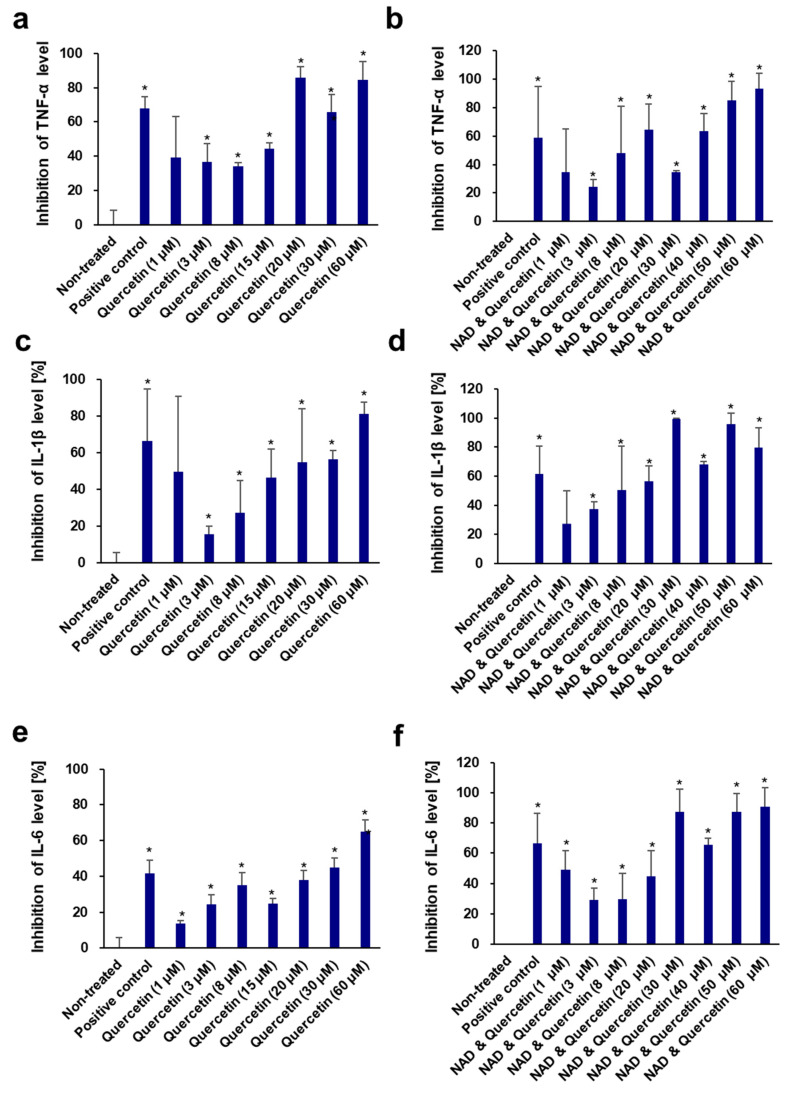
Dose-dependent effects of quercetin (**a**,**c**,**e**) and NAD^+^ and quercetin (**b**,**d**,**f**) on inhibition of pro-inflammatory cytokines TNF-α (**a**,**b**), IL-1β (**c**,**d**), and IL-6 (**e**,**f**) in LPS-induced RAW264.7 cells. Aminoguanidine hydrochloride was used as the positive control. NAD^+^ (70 μM) was added. All experiments were performed in triplicate. Student’s *t*-test was performed to determine significance between the non-treated group and test groups (* Significantly different results (*p* < 0.05)).

Very few studies have been conducted on the anti-inflammatory activity of NAD^+^. Tullius et al. reported that NAD^+^ could treat experimental autoimmune encephalomyelitis by promoting IL-10 production [113]. In this study, the anti-inflammatory activity of NAD^+^ was measured in vitro in LPS-induced RAW264.7 cells for the first time. NAD^+^, quercetin, and enoxolone showed no apparent toxic effects on RAW264.7 cells at concentrations ranging from 5 to 100 μM. NAD^+^ had only a weak inhibition (12.90–26.61%) of NO production at all these non-toxic concentrations. However, quercetin and enoxolone were found to possess significant NO inhibitory activity, with IC_50_ values of 3.36 ± 0.18 and 65.62 ± 0.44 μM, respectively (Figure 6), which were consistent with those reported in [114,115]. Nonetheless, we also tested the different combinations (NAD^+^ plus quercetin and NAD+ plus enoxolone) on the inhibitory effect of NO production. As a result, the corresponding IC_50_ values of quercetin and enoxolone were decreased to 0.85 ± 0.22 and 31.34 ± 7.66 μM after co-treatment with 70 μM NAD^+^ (Figure 7), indicating that NAD^+^ plus phytochemicals could synergistically exert anti-inflammatory activity.

**Figure 8:**
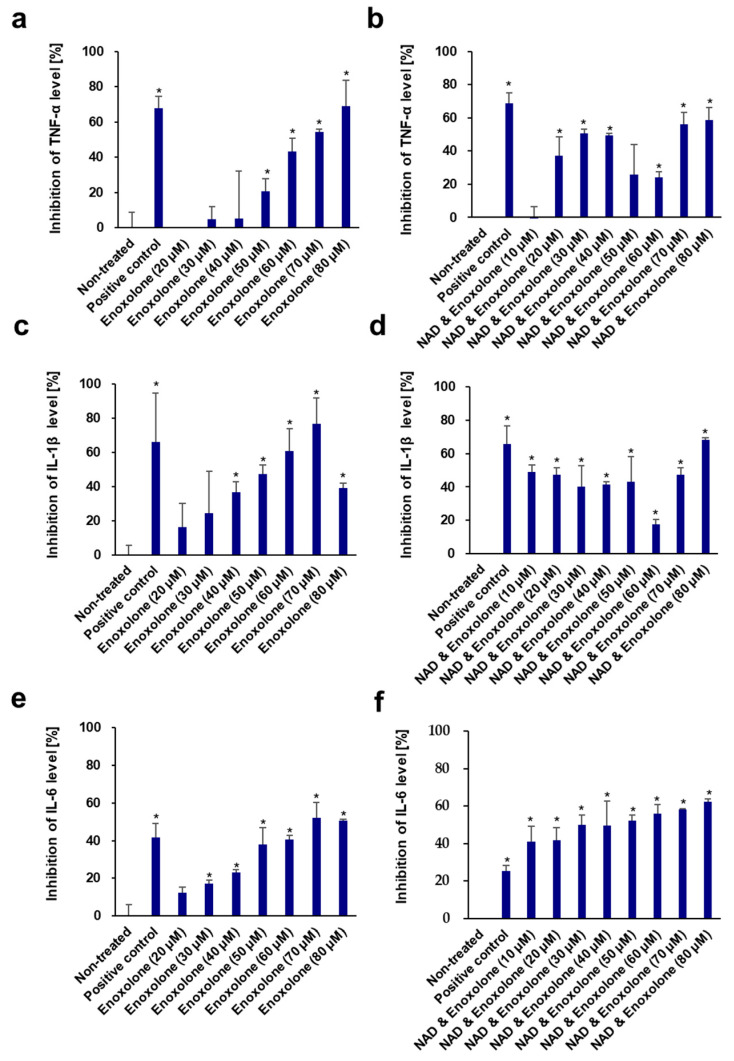
Dose-dependent effects of enoxolone (**a**,**c**,**e**) and NAD^+^ and enoxolone (**b**,**d**,**f**) on inhibition of pro-inflammatory cytokines TNF-α (**a**,**b**), IL-1β (**c**,**d**), and IL-6 (**e**,**f**) in LPS-induced RAW264.7 cells. Aminoguanidine hydrochloride was used as the positive control. NAD^+^ (70 μM) was added. All experiments were performed in triplicate. Student’s *t*-test was performed to determine significance between the non-treated group and test groups (* Significantly different results (*p* < 0.05)).

In addition, the inhibitory effects of quercetin and enoxolone against some pro-inflammatory cytokines (TNF-α, IL-1β, and IL-6) in LPS-induced RAW 264.7 cells were also measured (Figure 8). Quercetin displayed potent inhibition of TNF-α, IL-1β, and IL-6 production, with IC_50_ values of 7.02 ± 1.76 μM, 18.46 ± 7.66 μM, and 31.62 ± 10.31 μM, respectively. The IC_50_ values of quercetin were 10.01 ± 9.41 μM, 8.09 ± 7.24 μM, and 15.47 ± 10.73 μM, respectively, after co-treatment with 70 μM NAD^+^. Similarly, enoxolone also inhibited these three cytokines, with IC_50_ values of 66.42 ± 6.28 μM, 48.71 ± 8.01 μM, and 73.13 ± 5.17 μM, respectively. The IC_50_ values of enoxolone and NAD^+^ combinations on the inhibition of the three cytokines decreased to 45.64 ± 10.51 μM, 55.66 ± 9.84 μM, and 32.94 ± 11.49 μM, respectively. These results confirmed that the addition of these phytochemicals to the NAD^+^ complex could enhance anti-inflammatory activity and might regulate inflammaging.

## 4. Conclusions

NAD^+^, a crucial molecule in miscellaneous enzymatic reactions, orchestrates the entire cellular biological system and has been broadly studied for its actions and roles. Insights into the relationship between aging and NAD^+^ have triggered the use of NAD^+^ and its precursors for anti-aging purposes. Indeed, fragmented evidence from diverse experimental models and organisms has proven that the elevation of cellular NAD^+^ may slow or reverse the aging progress, not only at the in vitro level but also in vivo. However, whether this approach is also valid for treating skin aging has not been verified, which appears to be due to a lack of understanding of the mechanisms of NAD^+^ in skin cells. This study aimed to demonstrate the pharmacological efficacy of exogenous NAD^+^ on human fibroblasts and develop an approach to enhance its effectiveness by combining it with phytochemicals. Quercetin and enoxolone effectively inhibited CD38 expression in fibroblasts and elevated cellular NAD^+^ levels when exogenous NAD^+^ was added. Following in vitro experiments, we verified that exogenous NAD^+^ effectively counteracted UV-induced photoaging and intrinsic aging, which is associated with limited replicative reproduction. Consequently, this treatment extended the replicative lifespan of human fibroblasts, which also proved that the combination with the NAD^+^–boosting complex was more potent than NAD alone. Further investigation demonstrated that these protective effects against aging could be derived from enhanced autophagy and improved mitochondrial function. Our study provides new insights into the beneficial effects of NAD^+^ on the skin. In addition, our approach effectively enhanced the pharmacological efficacy of exogenous NAD^+^ treatment, addressing the unmet needs of both patients who require increased NAD^+^ in the skin for disease treatment and consumers who demand more effective and fundamental solutions for skin aging.

## Data Availability

The data that support the findings of this study are available from the corresponding author on request.

## Supplementary Materials

The following supporting information can be downloaded at: https://www.mdpi.com/article/10.3390/cells13211799/s1, Figure S1: Relative reactive oxygen species (ROS) generation; Figure S2: Western blot analysis for LC3; Figure S3: Wound scratch assay; Figure S4: mRNA expression for ACTG1, ARP2, and ARP3 analyzed by RT-qPCR; Table S1: Primer sequences for RT-qPCR.
