# Supplementary material for: Novel Approach to Skin Anti-Aging: Boosting Pharmacological Effects of Exogenous Nicotinamide Adenine Dinucleotide (NAD+) by Synergistic Inhibition of CD38 Expression
Source: Cells. 2024 Oct 30;13(21):1799. doi: 10.3390/cells13211799 (PMC11544843; doi:10.3390/cells13211799)
Supplement: Supplementary file 1 [file cells-13-01799-s001.zip › cells-3273718-supplementary.pdf]

cells

MDPI

Article

Novel approach for skin anti-aging: Boosting pharmacological effects of exogenous nicotinamide adenine dinucleotide by Synergistic Inhibition of CD38 Expression

(NAD+)

Seongsu Kang [1], Jiwon Park [1], Zhihong Cheng [2], Sanghyun Ye [1], Seung-Hyun Jun [1,*] and Nae-Gyu Kang [1,*]

1 LG Household and Health Care R&D Center, Seoul 07795, Korea
2 Department of Natural Medicine, School of Pharmacy, Fudan University, Shanghai 201203, China
* Correspondence: junsh@lghnh.com (S.-H.J.); ngkang@lghnh.com (N.-G.K.); Tel.: +82-2-6980-1239 (S.-H.J.); +82-2-6980-1533 (N.-G.K.)

Supplementary material

Genes	Forward (5' - 3')	Reverse (5' - 3')
CD38	TCTTGCCCAGACTGGAGAAAGG	TGGACCACATCACAGGCAGCTT
ULK1	GCAAGGACTCTTCCTGTGACAC	CCACTGCACATCAGGCTGTCTG
ATG5	GCAGATGGACAGTTGCACACAC	GAGGTGTTTCCAACATTGGCTCA
BECN1	CTGGACACTCAGCTCAACGTCA	CTCTAGTGCCAGCTCCTTTAGC
LC3B	GAGAAGCAGCTTCCTGTTCTGG	GTGTCCGTTCACCAACAGGAAG
P62	TGTGTAGCGTCTGCGAGGGAAA	AGTGTCCGTGTTTCACCTTCCG
TGF-b1	TACCTGAACCCGTGTTGCTCTC	GTTGCTGAGGTATCGCCAGGAA
PDGFB	GAGATGCTGAGTGACCACTCGA	GTCATGTTCAGGTCCAACTCGG
IL6	AGACAGCCACTCACCTCTTCAG	AGACAGCCACTCACCTCTTCAG
COL3A1	TGGTCTGCAAGGAATGCCTGGA	TCTTTCCCTGGGACACCATCAG
ATP5F1A	GCTCCTTACTCTGGCTGTTCCA	GCGGAGCAACAGAGACATCTGA
DRP1	CAAAGCAGTTTGCCTGTGGA	TCTTGGAGGACTATGGCAGC
PDK4	AACCGTATTTCTACTCGGATGCT	ACTCAAAGGCATCTTGGACCAC
OPA1	ACGTCTTTTGTCCAGCCTCT	GGTTAAAGCGCCCGTAACAT
ACTG1	CACCATTGGCAATGAGCGGTTC	AGGTCTTTGCGGATGTCCACGT
ARP2	GGTGTGACTCACATTTGCCCAG	TCAGCAGAGTGGTTGAAGGCGT
ARP3	TGCCTTAGCTGCATCTTGGACC	CTGCCAATCACATACCCTTCAGC

Supplementary Table S1. Primer sequences for RT-qPCR

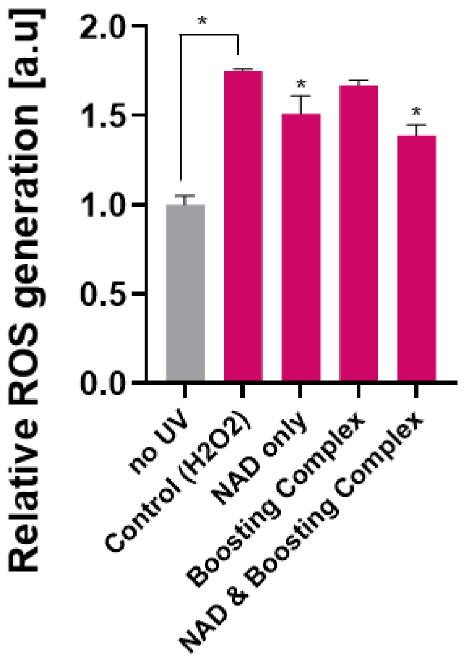

Supplementary Figure S1. Relative reactive oxygen species (ROS) generation. Cellular oxidative stress was estimated using dichlorofluorescin diacetate (DCFDA) assay. All experiments were performed in triplicate. Between negative control (no UV or no H_2O_2) and control group, Student's t-test was performed. One-way ANOVA (Dunnett's test) was performed for comparison between control and experimental groups. * Significantly different results ($p < 0.05$).

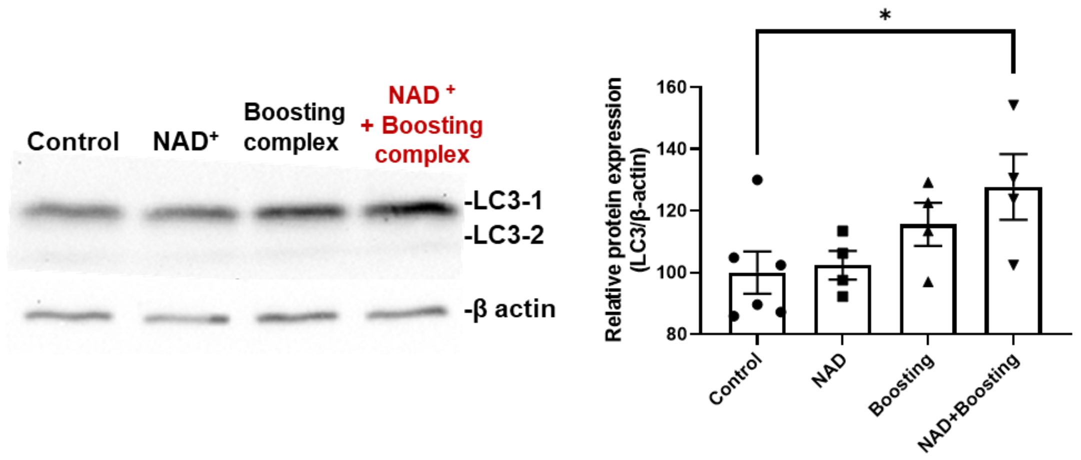

Supplementary Figure S2. Western blot analysis for LC3. In quantification, data are presented from independent experiments. Student's t-test was performed (* Significantly different results ($p < 0.05$)).

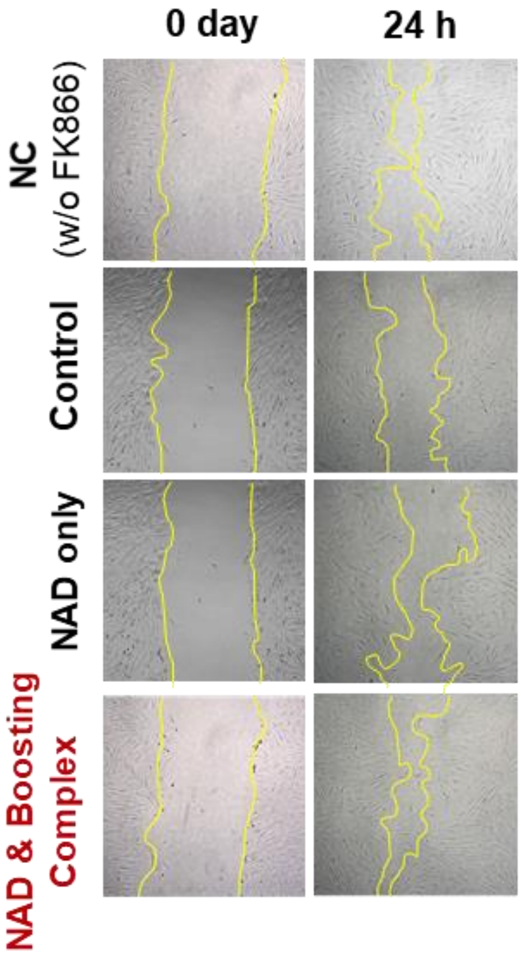

Supplementary Figure S3. Wound scratch assay. The wound area was measured after 24 h.

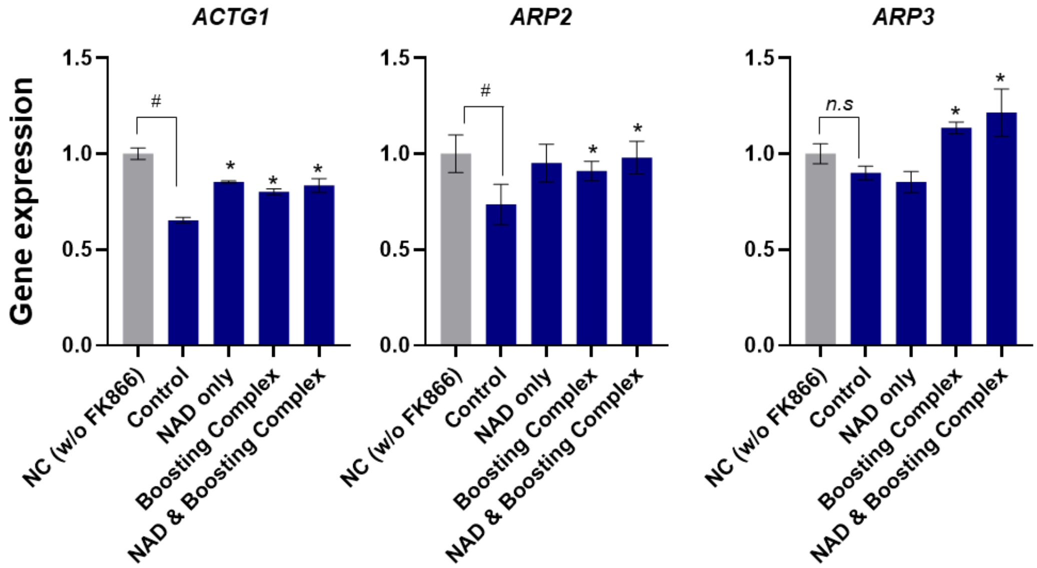

Supplementary Figure S4. mRNA expression for ACTG1, ARP2, and ARP3 analyzed by RT-qPCR. All experiments were performed in triplicate. Between negative control and control group, Student's t-test was performed (# Significantly different results ($p < 0.05$)). One-way ANOVA (Dunnett's test) was performed for comparison between control and experimental groups (* Significantly different results ($p < 0.05$)).

Disclaimer/Publisher's Note: The statements, opinions and data contained in all publications are solely those of the individual author(s) and contributor(s) and not of MDPI and/or the editor(s). MDPI and/or the editor(s) disclaim responsibility for any injury to people or property resulting from any ideas, methods, instructions or products referred to in the content.
